# Supplementary material for: Cadherin-11 contributes to the heterogenous and dynamic Wnt-Wnt-β-catenin pathway activation in Ewing sarcoma
Source: PLoS One. 2024 Jun 14;19(6):e0305490. doi: 10.1371/journal.pone.0305490 (PMC11178195; doi:10.1371/journal.pone.0305490)
Supplement: S1 File — (PDF) [file pone.0305490.s001.pdf]

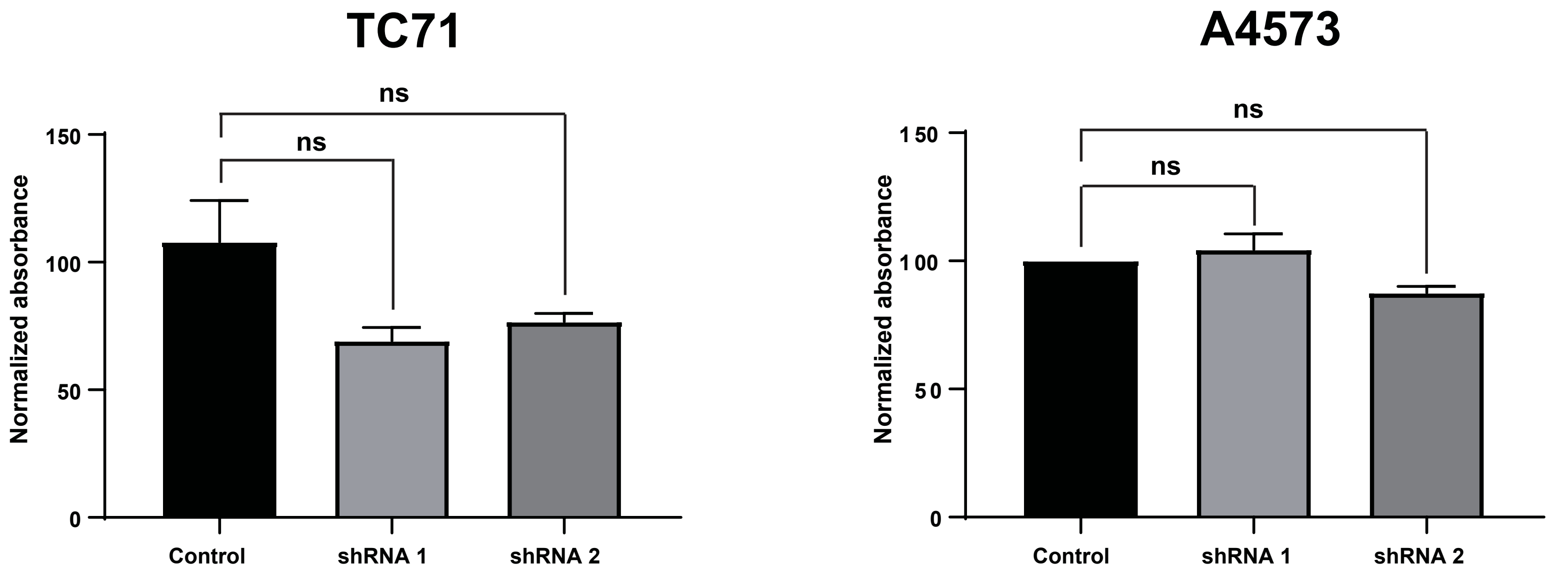

S1 Fig. β-Catenin knockdown does not affect cell proliferation. TC71 and A4573 cell proliferation was assessed, 48 hours after transfection with two separate shRNAs targting β-Catenin (shRNA1 and shRNA2), or shRNA targeting GFP (Control).

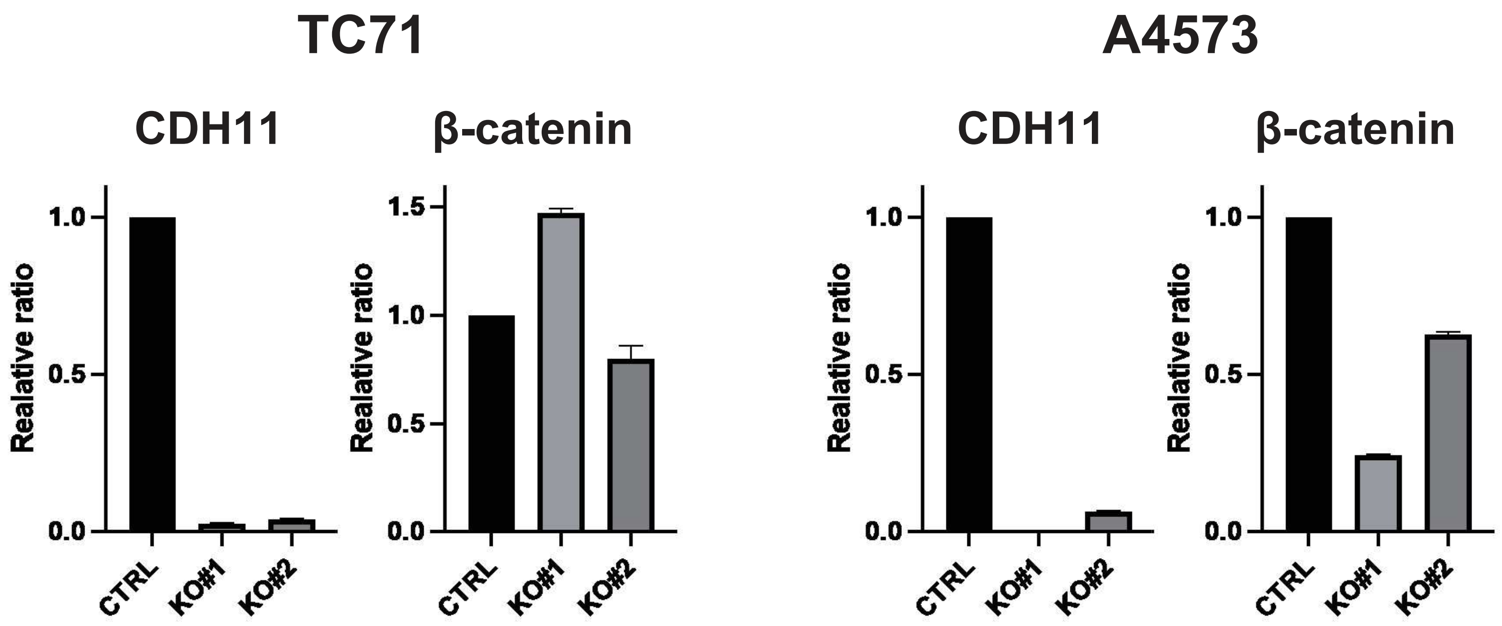

S2 Fig. CDH11 knockout does not lead to consistent down regulation of β-Catenin mRNA levels. β-Catenin mRNA expression evaluated by quantitative real-time PCR, CDH11 knockout did not lead to statistical difference of expression.

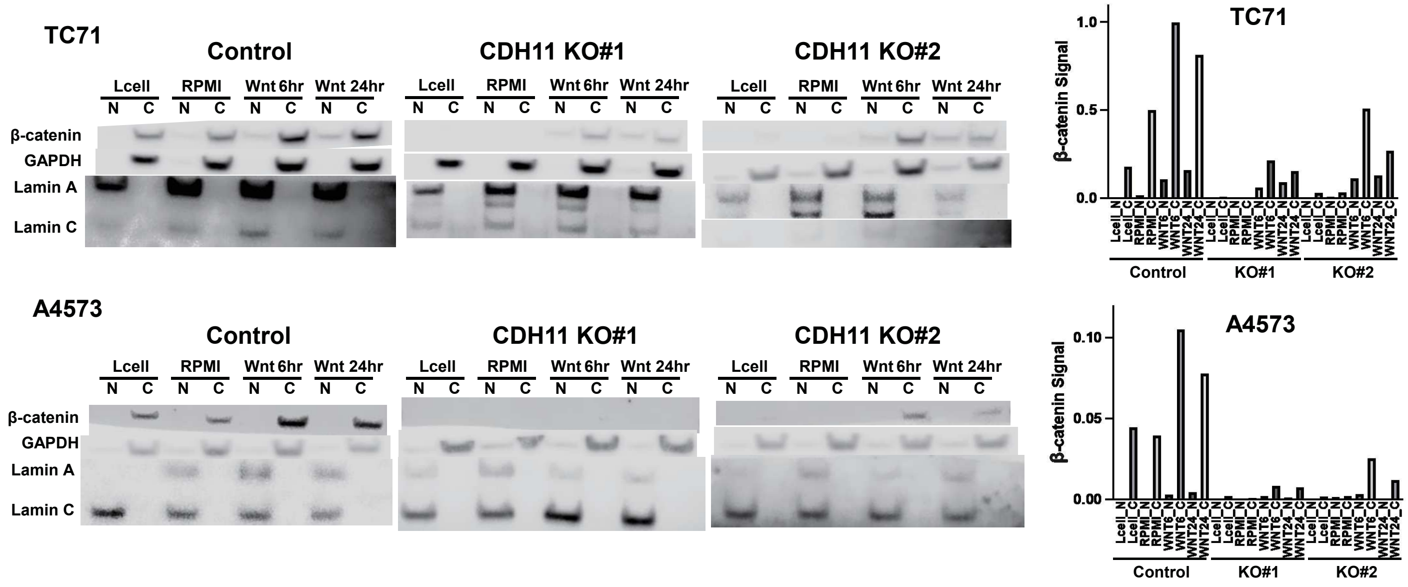

S3 Fig. Cell fractionation following Wnt3a stimulation in CDH11 knockout cells. TC71 and A4573 cells with CDH11 knockout, or transfected with an empty vector (control), were assessed for β-Catenin cellular localization through cell fractionation western blots. Cells were treated with control L cell media, or Wnt 3a media from Wnt3a expressing L cells , or regular culture media (RPMI). CDH11 knockout demonstrates general decrease in β-Catenin expression, but no evaluable change in nuclear β-Catenin expression.

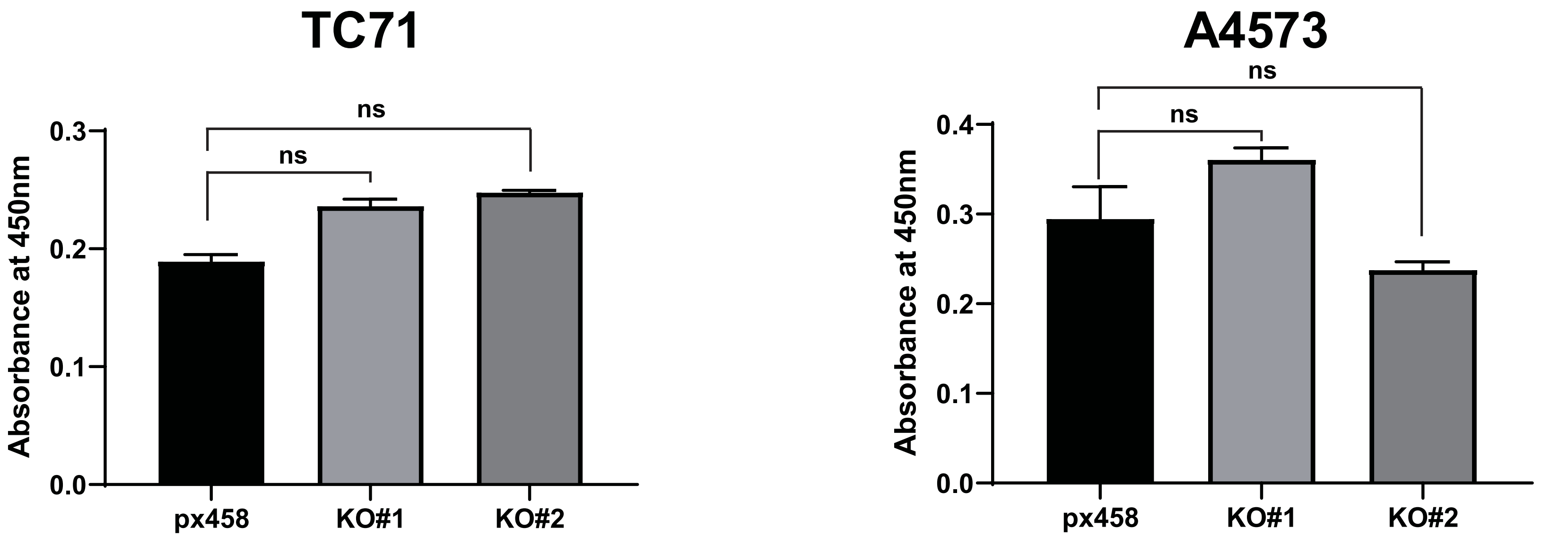

S4 Fig. CDH11 knockout does not affect cell proliferation. TC71 and A4573 cell proliferation was assessed, 48 hours after plating two separate CDH11 knockout clones (KO1#1 and KO#2), or empty px458 vector transduced cells as control (px458).
