# Supplementary material for: Cadherin-11 contributes to the heterogenous and dynamic Wnt-Wnt-β-catenin pathway activation in Ewing sarcoma
Source: PLoS One. 2024 Jun 14;19(6):e0305490. doi: 10.1371/journal.pone.0305490 (PMC11178195; doi:10.1371/journal.pone.0305490)

Fig1A

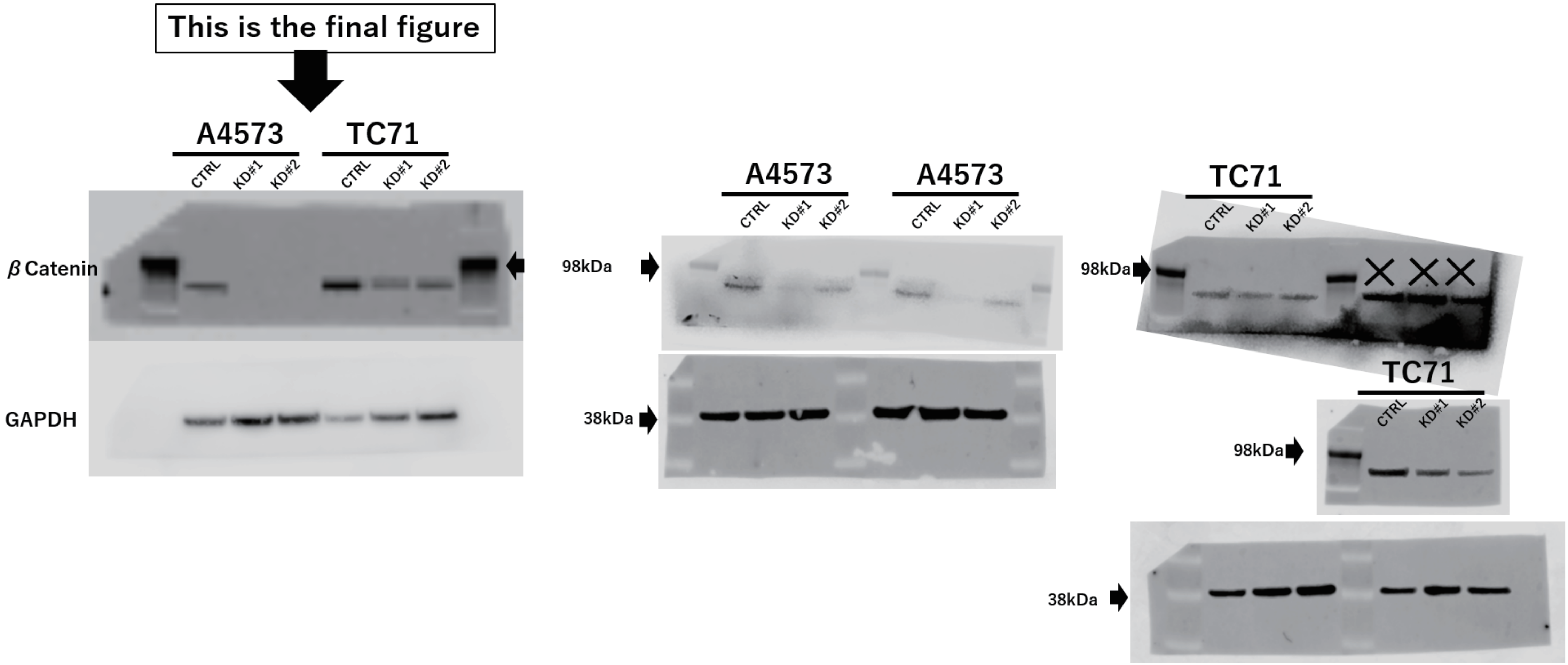

This is the final figure
A4573
TC71
CTRL
KD#1
KD#2
β Catenin
GAPDH
98kDa
38kDa
A4573
A4573
TC71
TC71
98kDa
38kDa

Fig3C

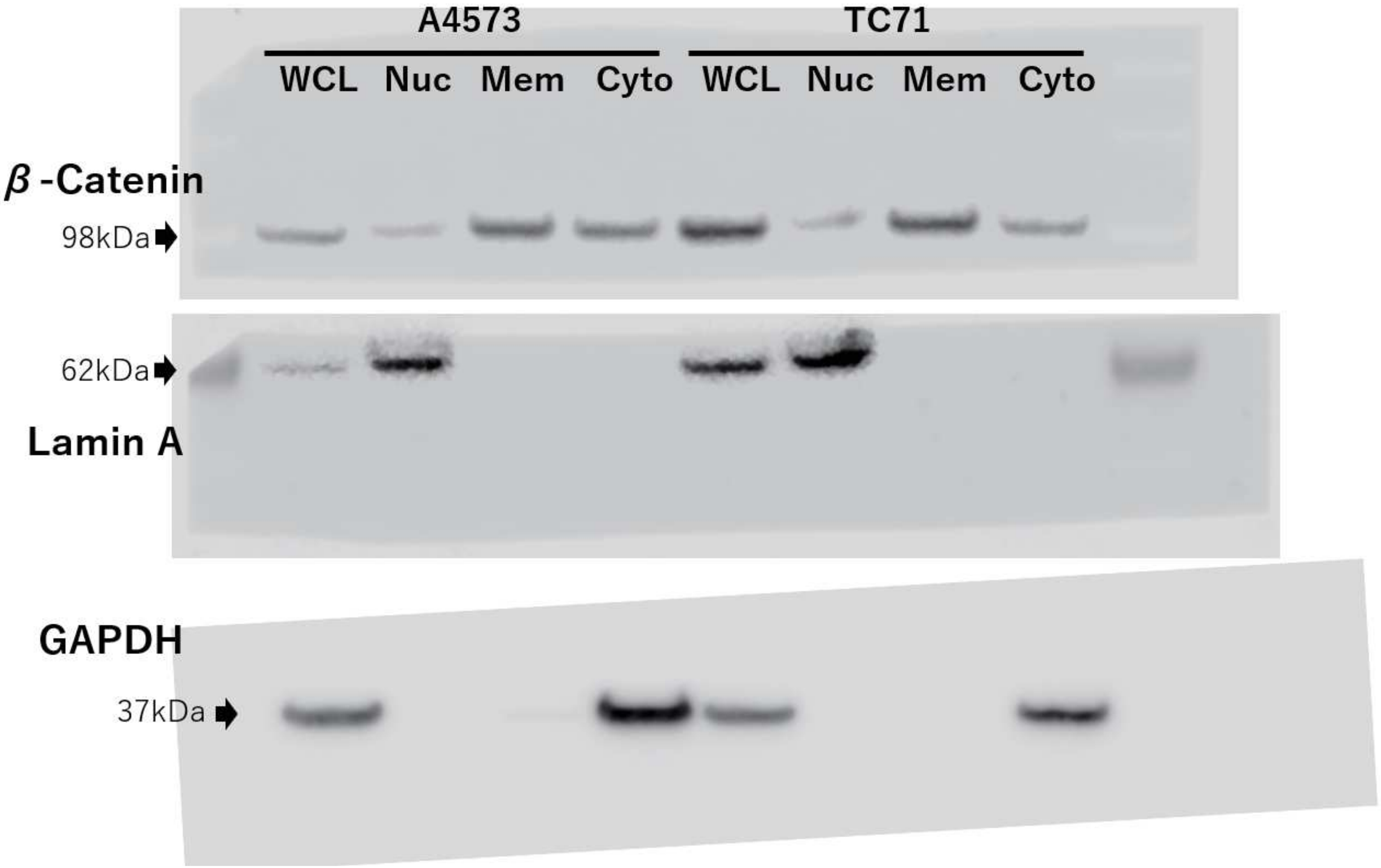

A4573
TC71
WCL
Nuc
Mem
Cyto
WCL
Nuc
Mem
Cyto
β -Catenin
98kDa
62kDa
Lamin A
GAPDH
37kDa

Fig4A

Those are the final figures.

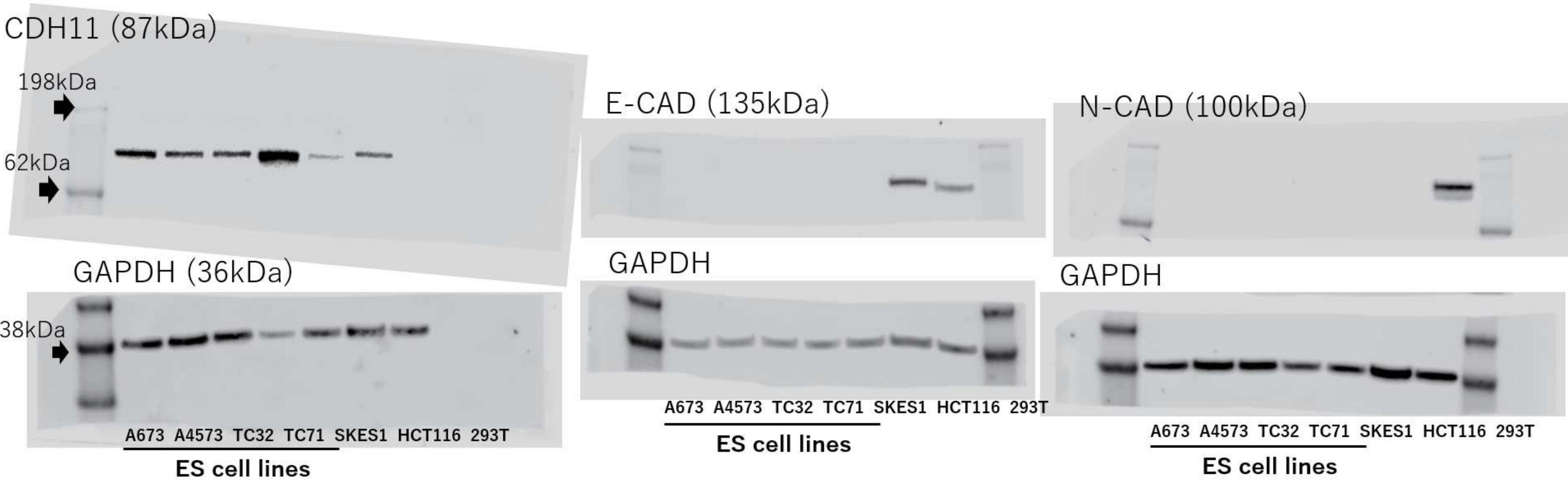

Fig4C

Those are the final figures.

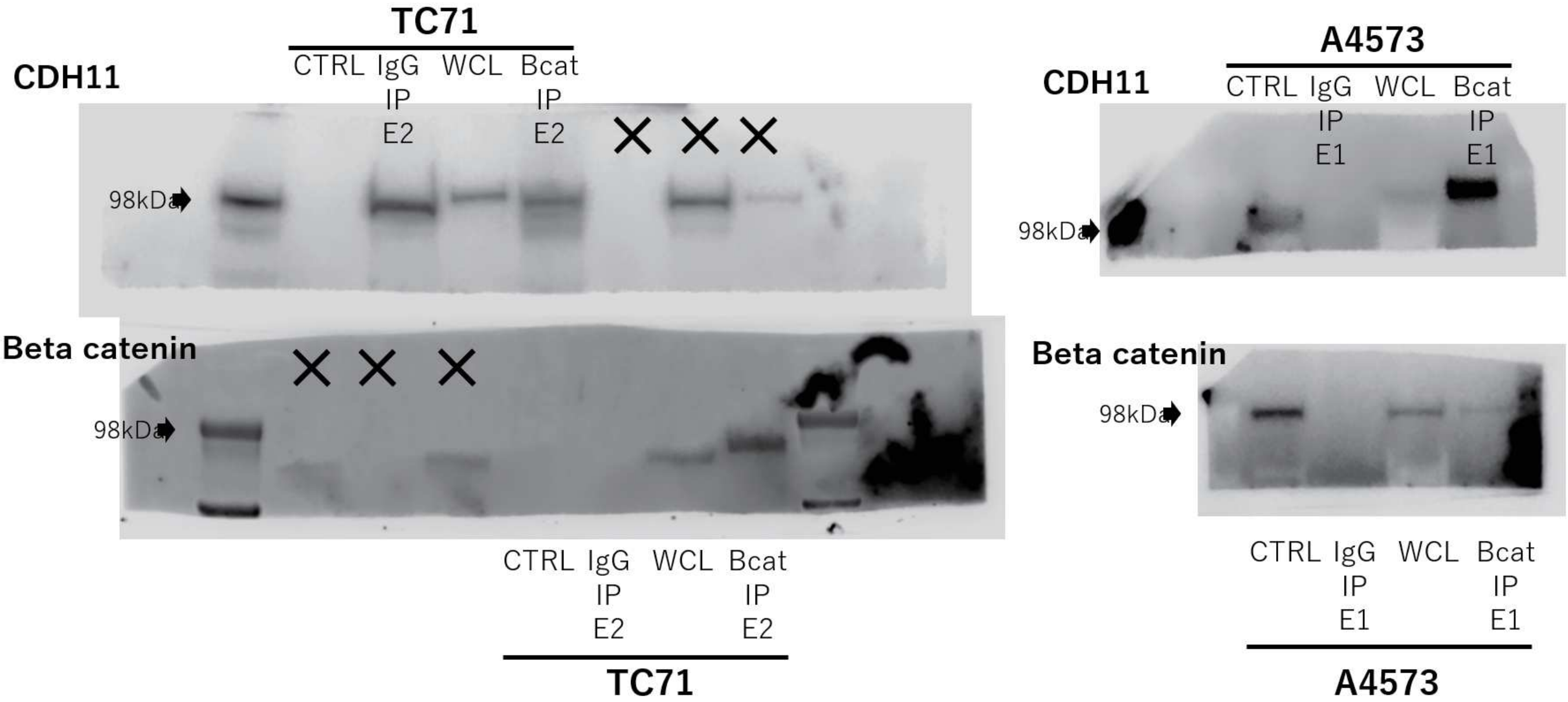

Fig5A

This is the final figure

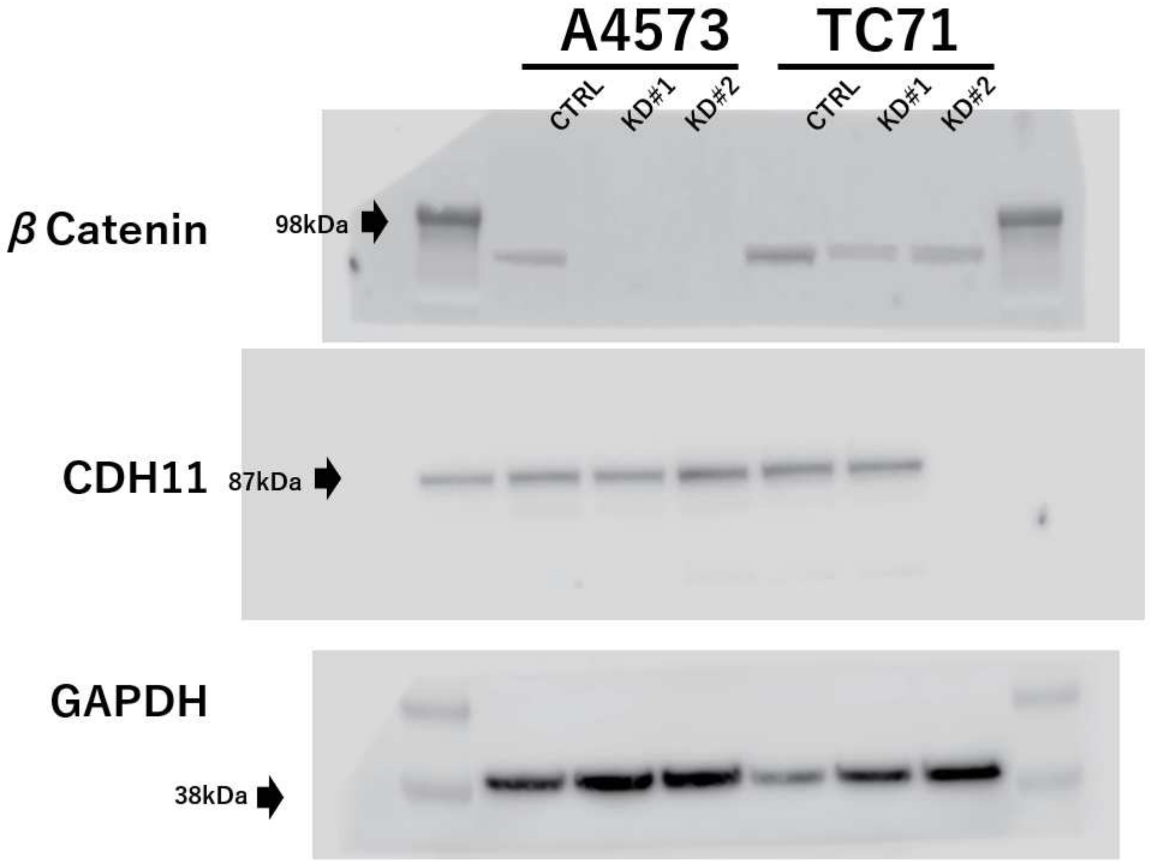

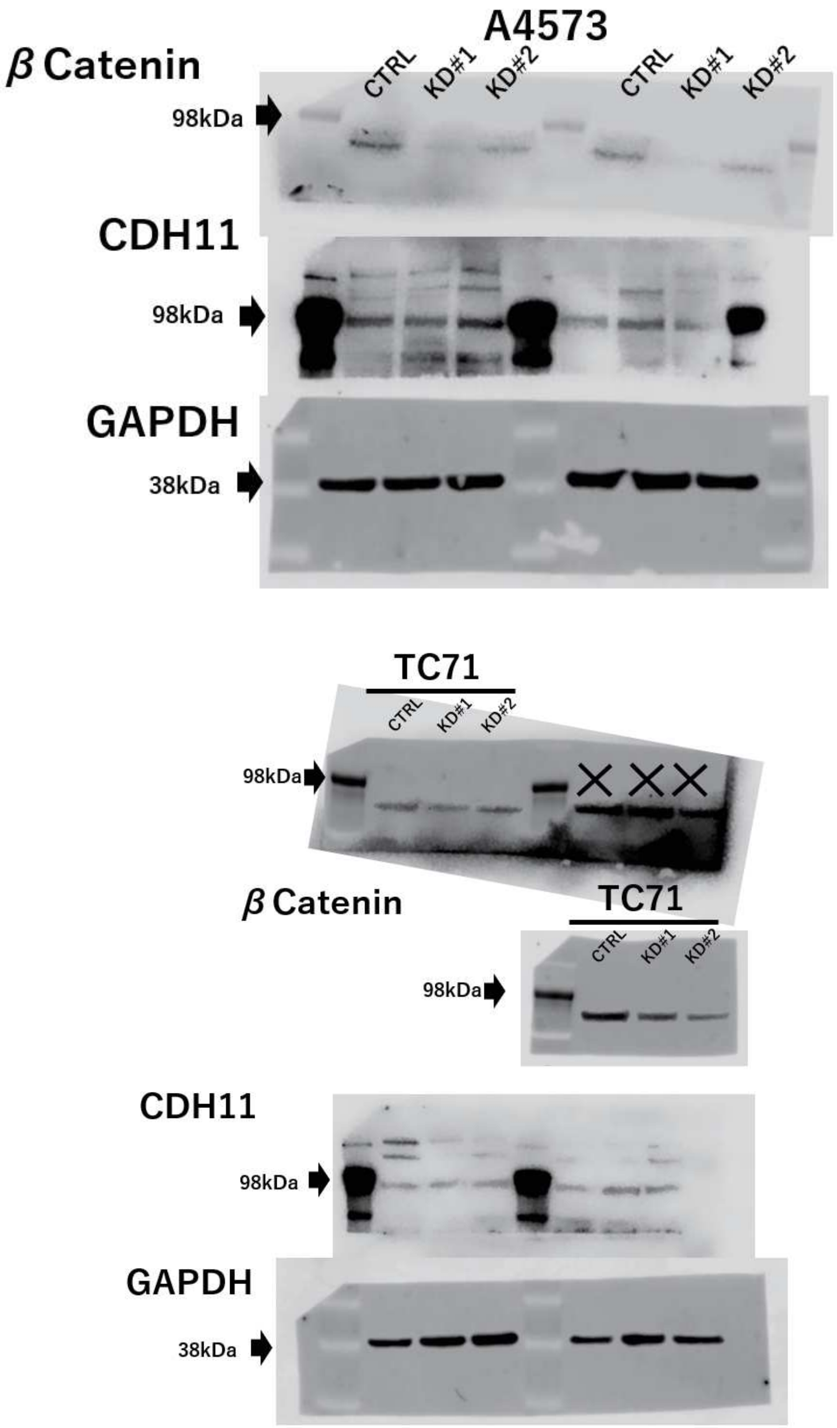

Fig5B

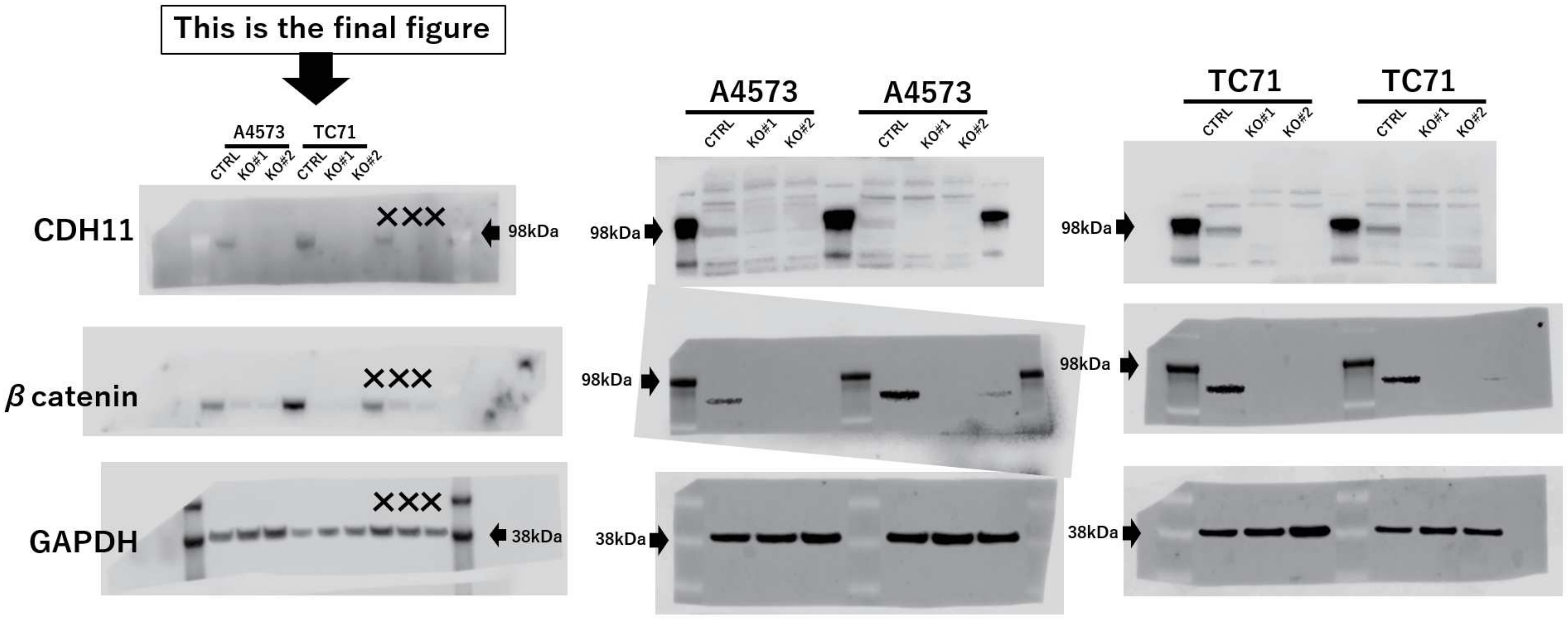
This is the final figure
A4573
TC71
CTRL
KO#1
KO#2
CDH11
β catenin
GAPDH
98kDa
38kDa
A4573
A4573
TC71
TC71

sFig3

Those are the final figures.

TC71

β-catenin(92kDa)

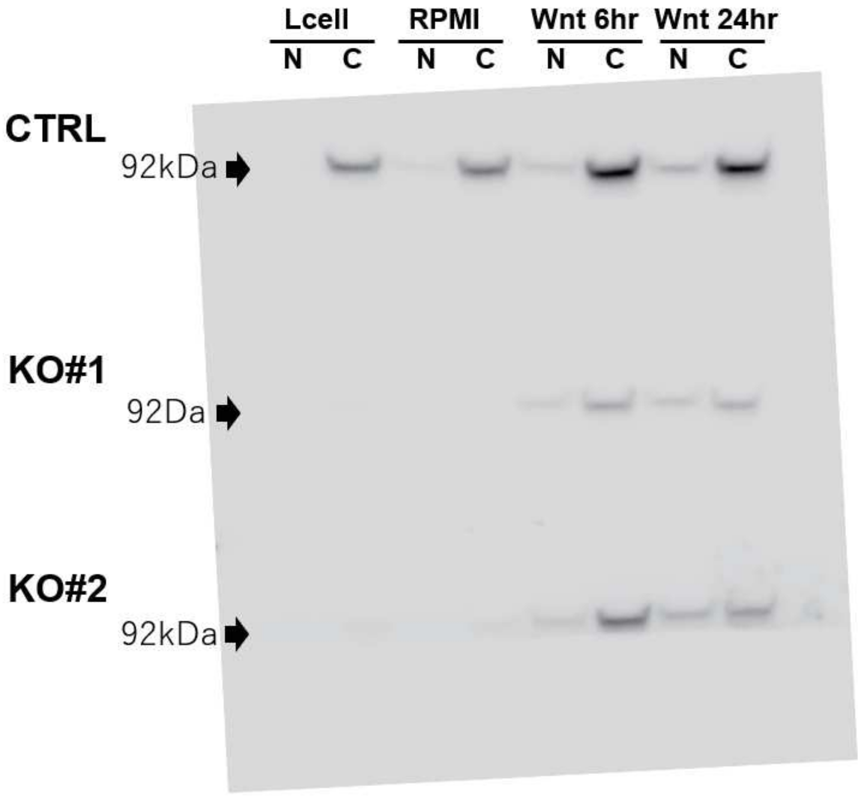

GAPDH(36kDa)

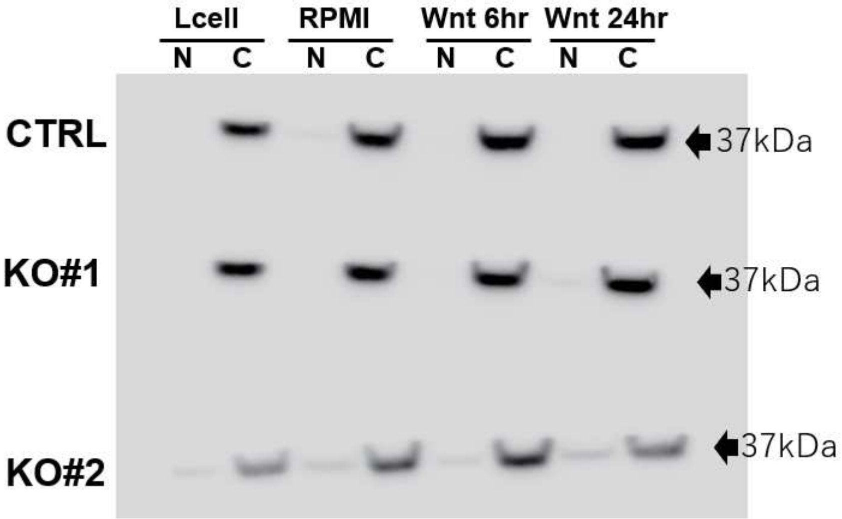

Lamin A/C(70kDa)

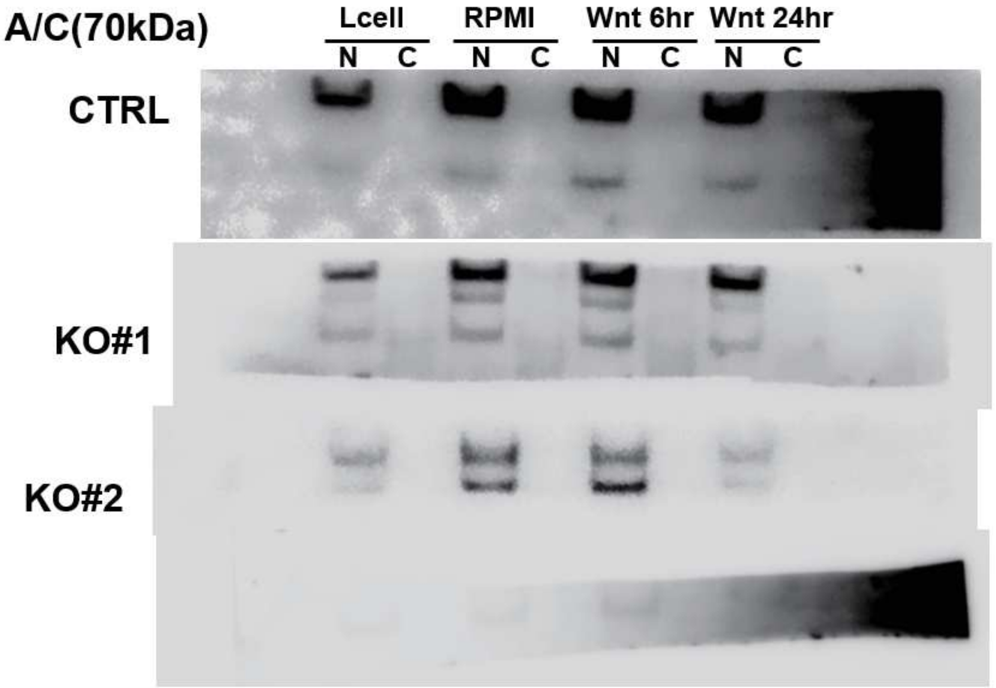

sFig3

Those are the final figures.

A4573

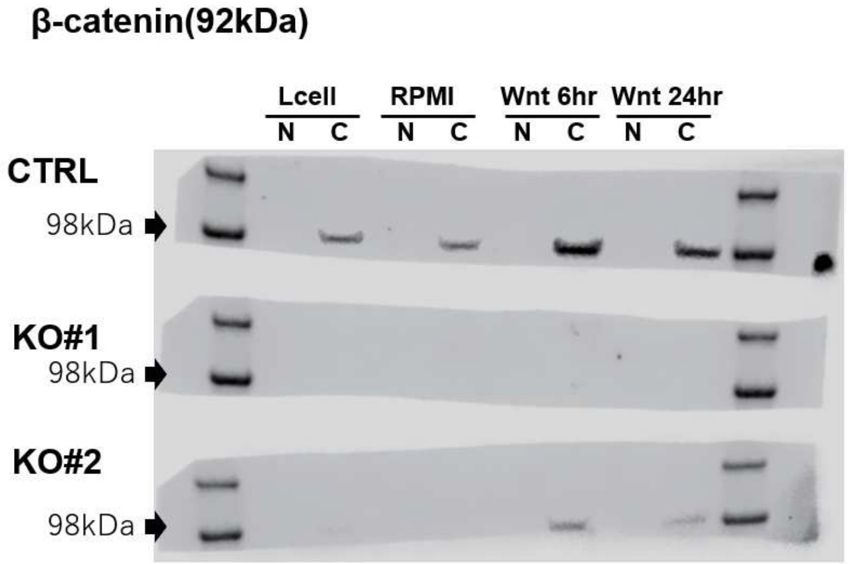

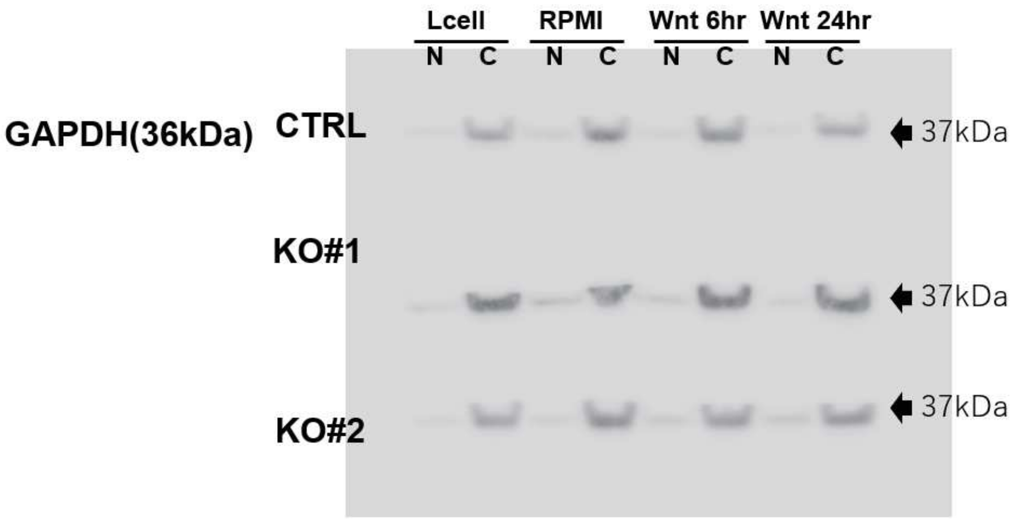

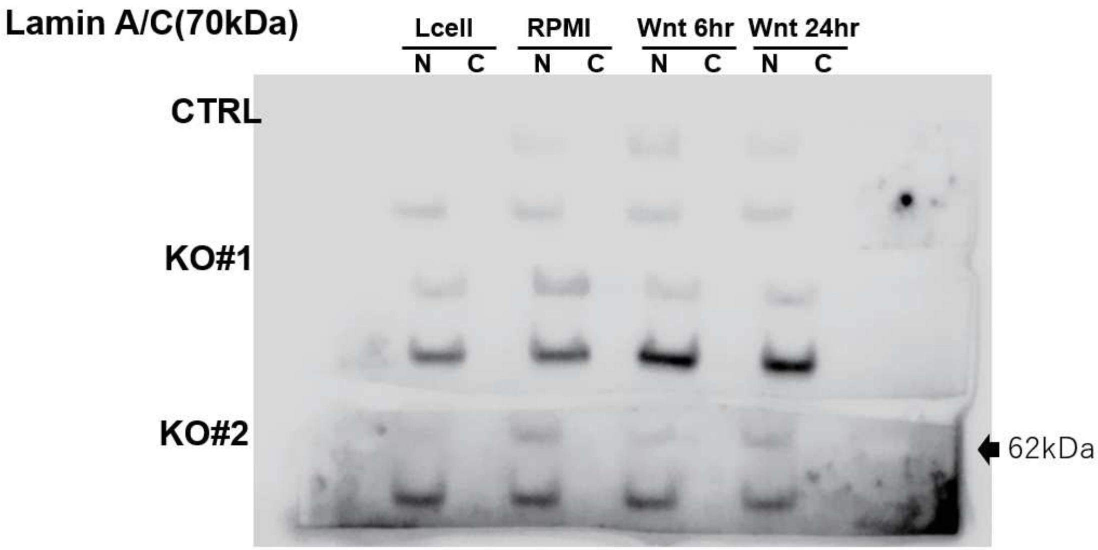

Supplement: S1 Raw images — (PDF) [file pone.0305490.s002.pdf]
