# Supplementary material for: Cadherin-11 contributes to the heterogenous and dynamic Wnt-Wnt-β-catenin pathway activation in Ewing sarcoma
Source: PLoS One. 2024 Jun 14;19(6):e0305490. doi: 10.1371/journal.pone.0305490 (PMC11178195; doi:10.1371/journal.pone.0305490)
Supplement: Attachment — Submitted filename: Response to Reviewers.docx [file pone.0305490.s003.docx]

We would like to thank the two reviewers for a thorough review and the valuable input from the initial review. Below, we have attached a point to point answer to the reviewer comments. With these revisions, we believe the manuscript has become significantly stronger. We hope you will agree that this report is now suitable for publication in PLOS ONE.

Sincerely,

Masanori Hayashi, MD

Editor comments:

When submitting your revision, we need you to address these additional requirements.

1. Please ensure that your manuscript meets PLOS ONE's style requirements, including those for file naming. The PLOS ONE style templates can be found at
   <https://journals.plos.org/plosone/s/file?id=wjVg/PLOSOne_formatting_sample_main_body.pdf> and 
   <https://journals.plos.org/plosone/s/file?id=ba62/PLOSOne_formatting_sample_title_authors_affiliations.pdf>

*Format has been corrected.*

2. To comply with PLOS ONE submissions requirements, in your Methods section, please provide additional information regarding the experiments involving animals and ensure you have included details on (1) methods of sacrifice, (2) methods of anesthesia and/or analgesia, and (3) efforts to alleviate suffering.

*This has now been added to the methods section, “Animal Experiments”.*

3. Note from Emily Chenette, Editor in Chief of PLOS ONE, and Iain Hrynaszkiewicz, Director of Open Research Solutions at PLOS: Did you know that depositing data in a repository is associated with up to a 25% citation advantage (https://doi.org/10.1371/journal.pone.0230416)? If you’ve not already done so, consider depositing your raw data in a repository to ensure your work is read, appreciated and cited by the largest possible audience. You’ll also earn an Accessible Data icon on your published paper if you deposit your data in any participating repository (<https://plos.org/open-science/open-data/#accessible-data>).

*The included single cell RNA-seq data will be deposited to GEO.*

4. Thank you for stating in your Funding Statement: 
"This work was supported by National Institutes of Health grant (K12HD068372) (M.H.) <https://www.nih.gov/grants-funding>, St. Baldrick’s Foundation Scholar Award (M.H.) <https://www.stbaldricks.org/for-researchers/,the> Hyundai Hope on Wheels Young Investigator Award (M.H.), and Tanabe-Bobrow Foundation award (M.H.) <https://hyundaihopeonwheels.org/>.  This study was partly supported by the National Institutes of Health P30CA046934 Bioinformatics and Biostatistics Shared Resource.
The funders had no role in study design, data collection and analysis, decision to publish, or preparation of the manuscript."

Please provide an amended statement that declares *all* the funding or sources of support (whether external or internal to your organization) received during this study, as detailed online in our guide for authors at <http://journals.plos.org/plosone/s/submit-now>.  Please also include the statement “There was no additional external funding received for this study.” in your updated Funding Statement. 
Please include your amended Funding Statement within your cover letter. We will change the online submission form on your behalf.

*We have included the amended funding statement within our new cover letter.*

5. In your Data Availability statement, you have not specified where the minimal data set underlying the results described in your manuscript can be found. PLOS defines a study's minimal data set as the underlying data used to reach the conclusions drawn in the manuscript and any additional data required to replicate the reported study findings in their entirety. All PLOS journals require that the minimal data set be made fully available. For more information about our data policy, please see <http://journals.plos.org/plosone/s/data-availability>.

Upon re-submitting your revised manuscript, please upload your study’s minimal underlying data set as either Supporting Information files or to a stable, public repository and include the relevant URLs, DOIs, or accession numbers within your revised cover letter. For a list of acceptable repositories, please see <http://journals.plos.org/plosone/s/data-availability#loc-recommended-repositories>. Any potentially identifying patient information must be fully anonymized.

Important: If there are ethical or legal restrictions to sharing your data publicly, please explain these restrictions in detail. Please see our guidelines for more information on what we consider unacceptable restrictions to publicly sharing data: <http://journals.plos.org/plosone/s/data-availability#loc-unacceptable-data-access-restrictions>. Note that it is not acceptable for the authors to be the sole named individuals responsible for ensuring data access.

We will update your Data Availability statement to reflect the information you provide in your cover letter.

*Data Availability has been updated in the cover letter. All blots are now uploaded as S1 Raw, and genomic data has been uploaded to GEO.*

6. PLOS ONE now requires that authors provide the original uncropped and unadjusted images underlying all blot or gel results reported in a submission’s figures or Supporting Information files. This policy and the journal’s other requirements for blot/gel reporting and figure preparation are described in detail at <https://journals.plos.org/plosone/s/figures#loc-blot-and-gel-reporting-requirements> and <https://journals.plos.org/plosone/s/figures#loc-preparing-figures-from-image-files>. When you submit your revised manuscript, please ensure that your figures adhere fully to these guidelines and provide the original underlying images for all blot or gel data reported in your submission. See the following link for instructions on providing the original image data: <https://journals.plos.org/plosone/s/figures#loc-original-images-for-blots-and-gels>. 
  
In your cover letter, please note whether your blot/gel image data are in Supporting Information or posted at a public data repository, provide the repository URL if relevant, and provide specific details as to which raw blot/gel images, if any, are not available. Email us at plosone@plos.org if you have any questions.

This has been clarified. Raw blot data has been submitted as a supplemental file.

7. Please include your full ethics statement in the ‘Methods’ section of your manuscript file. In your statement, please include the full name of the IRB or ethics committee who approved or waived your study, as well as whether or not you obtained informed written or verbal consent. If consent was waived for your study, please include this information in your statement as well.

*We included a statement that the animal studies were approved by our local IACUC. No human samples were used, so no disclosure for IRB.*

Additional Editor Comments:

Both reviewers find your study interesting , but have raised a number of relevant points that needs to be addressed point by point. I would be happy to grant extra time should this be needed.

Reviewer comments:

Reviewer #1: The study by Shirai et al. aimed to investigate the contribution of

CDH11 and b-catenin in Ewing sarcoma. This work could have been interesting; however, it lacks rigor and several points need to be clarified:

1. The English and scientific writing could be much improved.
In addition, the figure legends lack information that is important to understand the figures.
References need to be reformatted (e.g., #39 taking half a page!).

*The main document was proofread by a native English speaker and all sections have been significantly modified. Figure legends were revised with further details. In our original submission, we had used Endnote to format the references according to the PLOS template. For reference 39, we found that Endnote will automatically format this reference in this fashion due to a formatting requirement that appears to force all the COI disclosures to be included. We could not find any other way to format this reference, so we removed this and replaced it with a similar report that can still emphasize the same point.*

2. In general, blots and immunostaining should be quantified.
*All western blots were quantified using Image Studio Ver5.2 from LI-COR and immunostaining images were quantified using BZ-X710 from Keyence.*

3. Fig 1: Good expression of b-catenin is still observed in TC71 cells after knockdown (A). So, how the authors can explain a such dramatic effect in the following experiments (like in B&C)? Especially given that basic b-catenin signaling is very low in these cells.
*This is an excellent point, considering the relatively heterogeneous expression of β-Catenin in these cells. We do not have an exact explanation on why colony formation and soft agar formation is so dramatically decreased. However, to maintain scientific objectivity, we also never make any claim that the degree of decrease in Fig 1B-C is nothing more than statistically significant. We speculate that even the degree of knockdown accomplished in Fig 1A is enough to significantly decrease the colony formation and soft agar formation, because a very small number of cells within the general population is Wnt/β-Catenin active at a time (as shown in Fig 2), and these cells are critical to the colony formation.*

4. Fig 2: heterogeneity of Wnt/b-catenin activation in Ewing sarcoma is already published (reference #20 cited by the authors), and this figure does not bring any new information and should be removed. Moreover, following the methods described, the conclusions driven from this figure are biased: a) The whole A4573 xenograft has been used for the scRNAseq, so this includes mouse cells that have infiltrated the tumor. b) How Wnt signaling can be enriched when it is activated in only 4.3% of the A4573 population in vivo? c) It was performed on A4573 only, this is not representative.
It would have been more interesting to perform scRNAseq on CDH11 KO/WT cells.

*In the original Pedersen work that we cited, Wnt/β-Catenin activation heterogeneity was only demonstrated in cell cultures, and in vivo analysis was limited to IHC of β-Catenin in archival tumors. We included this figure due to two reasons. First, our work contains mostly in vivo results in a mouse model, demonstrating heterogeneity of Wnt/β-Catenin signaling, a finding that Pedersen did not demonstrate. Second, even if one paper has published a similar figure in the past using a different cell line, we feel that demonstrating that we can reproduce some of Pedersen’s conclusions (Fig2A is the only figure that is close to Pedersen) is important for scientific rigor. To avoid confusion, we have emphasized in the new version that we are merely corroborating published data for Fig2A, and that the new addition to literature is the findings in vivo. In figure2B, we demonstrate that 4.3% of tumor cells were activated (representative figure), indicating a small subpopulation of activated cells exist in vivo. We have done this experiment in triplicate, and the data is now demonstrated as a comparison to baseline activation in vitro that was observed in Fig 1A. This is why we conclude there is heterogeneity in Wnt activation within the tumor. In figure B-E, our original intention was to possibly do this in two cell lines. However, the single RNA-seq experiment for the TC71 derived xenograft did not pass our QC. Violin plot shown below. Considering the significant cost of single RNA-seq, and the fact that this figure is not the main point of the paper, we chose to only include A4573 xenograft data. Considering the criticism of only having one cell line, we have significantly toned down the discussion and results.*

*As standard QC, we remove all cells from the xenograft scRNA-seq data that aligned to the mouse transcriptome, and the data shown are from all human cells that have passed QC.*

5.Fig 4: IP (C) should be repeated in the other way, i.e., with CDH11 IP. The authors mentioned in the text that CDH11 interacted with "non-phosphorylated" b-catenin, but without showing it.
The immunostaining (B) is not convincing, several cells are negative; CDH11 antibody does not look very good.

*We have attempted CDH11 IP, however this was technically difficult due to a lack of a robust enough antibody. We have removed the text mentioning non-phosphorylated β-catenin. The immunostaining was revised according to comments from reviewer 2 using different secondary antibodies. In our new experiment, we used an Alexa Fluor 488 conjugate secondary antibody for β-Catenin, and Alexa Fluor 647 conjugate secondary antibody for CDH11, and the immunostain is much more robust.*

6. Fig 5: Same comment as for Fig 4, immunostainings are not convincing (F): A4573 showed no expression of b-catenin in the control; we do not see any differences in nuclear localization of b-catenin between the different conditions in TC71 and A4573 cells (quantification is necessary). In addition, WB did not show any b-catenin translocation to nucleus in A4573 after Wnt stimulation (E) in contrast to what it should.

*We have removed the immunostains included in the previous Fig 5 B D and F. As other figures in the paper, we have now quantified the western blot imaging. For the previous Figure 5E (now in supplemental), the sensitivity of western blots in detecting protein from a small subpopulation is so poor that we feel it did not prove nor disprove the hypothesis. Therefore, we moved this figure to supplemental, and have noted such in the main text. For the previous Fig 5F (now Fig 5C), we have added statistical analysis.*

7. Fig 6: In C, counting cells with neurite outgrowth is a bit fishy (on pictures we can clearly see some in all conditions), it would have been better to quantify cell area.
In the cell migration assay (D), It is tricky to associate CDH11 to b-catenin signaling, since in basal condition this pathway is activated in only few cells (and here the control showed good migration). What is the status of b-catenin signaling during cell migration? The assay should be repeated including stimulation with Wnt.
Another type of cell migration like a wound assay could be considered.

*In Fig 6C, we have now quantified the cell area, and performed statistical analysis as recommended. We agree that claiming that Fig 6 proves that CDH11 knockout leading to decreased β-Catenin signaling activation is an over-representation of the data, and we have amended the text to tone it down. We have attempted Wnt stimulation experiments for cell migration, but we have faced significant technical difficulties due to the background of the system we are using. We have been performing Wnt ligand stimulation using a Wnt3a secreting L-cell system. In this system, the Wnt3a media (or control L-Cell media), is collected as a supernatant from these cells after incubation. This supernatant is often nutrient poor (since it has been used by L-cells for 48-96 hours), DMEM based (All of our Ewing sarcoma cells are cultured in RPMI), and Ewing sarcoma cell lines do not grow normally in these media. Which is why we have limited stimulation experiments to Fig2A and Fig 5, where we only quantify the β-catenin signaling subpopulation after Wnt stimulation. When we used this nutrient poor DMEM based supernatant and tried to perform migration experiments, the cells did not migrate enough even at baseline to have any measurable statistical change. Because of this limitation, we were unsuccessful in repeating the assay with Wnt stimulation.*

8. Fig 7: Using CRISPR/Cas9, the authors have knock-out CDH11 (so the cells do not express it anymore, as seen in the blot 5C; knock-out is different than knock-down using shRNA, where in the latest some expression can still be observed). So why is there huge discrepancies between KO#1 and 2 in 7A and B (in the two cell type??)? In this context, the results does not look reliable.
To what correspond the control? Has flow cytometry analysis been performed for CDH11 expression at the cell surface?

*We have performed flow cytometry to demonstrate CDH11 cell surface expression was depleted in knockout conditions, in Fig 5, where we initially demonstrate successful knockout. The conclusion of Fig 7A and 7B has been clarified, since they are distinct experiments. Fig 7A was quantifying lung metastasis using ddPCR, a much more sensitive and specific measurement of metastasis. Fig 7B is enumerating liver metastases visually, which is a less sensitive method of metastasis quantification. Additionally, human patients rarely get liver mets, and liver mets are only found in this particular mouse model. While we personally do not feel strongly about liver mets, we listed Fig 7B because readers who are familiar with this experiment will be curious about what happened. We speculate that the variability found between knockout 1 and 2 is multifactorial, as often is the case for preclinical in vivo experiments. This is evident in the control arm of this experiment where metastasis burden is widely variable, a result that we commonly see in all of our experiments, and even more so in clinical trials. The control is a TC71 or A4573 cell line transfected with an empty px458, which has now been clarified in the method section and main text.*

Reviewer #2: The manuscript examines the role of Cadherin-11 in Wnt/β-Catenin signalling in Ewing sarcoma (ES) cells. The authors suggest that cadherin-11 contributes to Wnt/β-Catenin signalling in ES cells by controlling the expression of β-catenin. Using a xenograft model, the authors suggest that loss of cadherin-11 in ES cells can lead to a less metastatic phenotype. In general, the manuscript is technically sound. Although, some of the claims are supported with the experimental data, some major issues remain to be addressed.
Major comments:
1. The results showing the reduction in the protein levels of β-catenin expression in Cadherin-11 KO cells in interesting. In this context, mRNA levels of β-catenin and Cadherin-11 would help to know if the reduction was due to degradation or at transcription level. Authors could also use proteasomal inhibitors MG-132 to demonstrate the role of Cadherin-11 in the regulation or stabilization of β-catenin expression.

*This is an excellent point. In response, we have performed an RT-qPCR assay to assess for mRNA levels of β-Catenin and CDH11 in the knockout cells., and this has been included in S2 Fig. Here, we found that the mRNA level of β-catenin was reduced in CDH11 knockout A4573 cells, but not one of the CDH11 knockout TC71 cells. This may indicate the mechanism that CDH11 is mainly contributing to β-Catenin protein stability. We agree that using a proteasomal inhibitor such as MG-132 to differentiate between regulation and stabilization would be interesting. However, MG-132 treatment has been reported to significantly alter EWS-FLI1 degradation in Ewing sarcoma cells (PMID 27875302), and we are concerned that this will significantly mask any other change that will be observed in the cells.*

2. The data set in figure 5 could improve with quantification and statistical analysis. Importantly, the nuclear-cytoplasmic ratio of β-catenin in the western blots would help to understand if the reduced nuclear β-catenin in cadherin-11 KO cells are due to reduced total expression of β-catenin or due to changes in its localization.

*In Fig 5A-B, we have now added quantification and statistical analysis of the western blots. For Fig 5D we have added statistical analysis of the ratio of GFP positive canonical Wnt active cells. For nuclear-cytoplasmic ratios of β-Catenin in Fig 5C, we quantified the western blots to attempt to perform statistical analysis. However, due to the nature of measuring a lowly expressed protein using western blots, the quantified ratio was too variable to compare. Therefore, Fig 5C has now been moved to supplemental, and the main text has been altered to clarify that no statistical significance was observed.*

3. The immunofluorescence staining of Cadherin-11 throughout the article is not very convincing to evaluate the expression level. Protein levels of Cadherin-11 in CRISPR-based Cadherin-11 KO cells would be better than immunofluorescence.

We agree that the original immunofluorescence imaging was not robust enough to evaluate the expression level of CDH11. We have reimaged the immunofluorescence images with revised secondary antibodies using Alexa Fluor 488 and 647. With improved imaging conditions, Figure 4B and Fig 7D now contain immunofluorescence imaging.

Minor comments:
1. The authors state that β-catenin is predominantly localized in the cell membrane. The fractions used for western blotting are cytoplasmic and not purely membrane fractions. Addition of Wnt ligands in experiments related to 3C would be helpful to know if β-catenin is predominantly localized in the cytoplasmic fractions despite activation of Wnt/β-Catenin signalling. Based on figure 2A, it seems there is very little activation of Wnt/β-Catenin signalling in the absence of Wnt ligands and this could explain the reduced nuclear localization of β-Catenin

*This is an excellent point. To demonstrate specific localization within the cell, we performed additional cell fractionization experiments shown in Fig 3C. Here, we demonstrate that β-Catenin localizes most commonly in the membranous fraction, followed by the cytoplasmic fraction. The main text has been altered according to this result.*

2. For immunofluorescence the combination of 488 and 647 would be better instead of 488 and 555 to avoid cross-excitation issues mainly for cadherin-11 staining.

*Thank you for this suggestion. As suggested, 488 and 555 were exchanged into 488 and 647, which produced significantly improved imaging.*

3. Figure legends are not completely clear enough to understand the data. For example, it is not clear what is Lcell and RPMI. Also, in figure 5C its mentioned as CDH11 KO but mentioned as CDH11 KD in 5E.

*The description of figure legends were revised appropriately to include complete information, such as explanation of abbreviations.*
